# Downregulation of Keratin 76 Expression during Oral Carcinogenesis of Human, Hamster and Mouse

**DOI:** 10.1371/journal.pone.0070688

**Published:** 2013-07-30

**Authors:** Srikant Ambatipudi, Priyanka G. Bhosale, Emma Heath, Manishkumar Pandey, Gaurav Kumar, Shubhada Kane, Asawari Patil, Girish B. Maru, Rajiv S. Desai, Fiona M. Watt, Manoj B. Mahimkar

**Affiliations:** 1 Cancer Research Institute, Advanced Centre for Treatment, Research and Education in Cancer, Tata Memorial Centre, Navi Mumbai, India; 2 King’s College London Centre for Stem Cells and Regenerative Medicine, London, United Kingdom; 3 Department of Pathology, Tata Memorial Hospital, Tata Memorial Centre, Parel, Mumbai, India; 4 Department of Oral Pathology and Microbiology, Nair Hospital Dental College, Mumbai, India; Sapporo Medical University, Japan

## Abstract

**Background:**

Keratins are structural marker proteins with tissue specific expression; however, recent reports indicate their involvement in cancer progression. Previous study from our lab revealed deregulation of many genes related to structural molecular integrity including KRT76. Here we evaluate the role of *KRT76* downregulation in oral precancer and cancer development.

**Methods:**

We evaluated KRT*76* expression by qRT-PCR in normal and tumor tissues of the oral cavity. We also analyzed K76 expression by immunohistochemistry in normal, oral precancerous lesion (OPL), oral squamous cell carcinoma (OSCC) and in hamster model of oral carcinogenesis. Further, functional implication of KRT76 loss was confirmed using KRT76-knockout (KO) mice.

**Results:**

We observed a strong association of reduced K76 expression with increased risk of OPL and OSCC development. The buccal epithelium of DMBA treated hamsters showed a similar trend. Oral cavity of KRT76-KO mice showed preneoplastic changes in the gingivobuccal epithelium while no pathological changes were observed in KRT76 negative tissues such as tongue.

**Conclusion:**

The present study demonstrates loss of KRT76 in oral carcinogenesis. The KRT76-KO mice data underlines the potential of *KRT76* being an early event although this loss is not sufficient to drive the development of oral cancers. Thus, future studies to investigate the contributing role of KRT76 in light of other tumor driving events are warranted.

## Introduction

Keratins are filament forming proteins of epithelial cells and are essential for normal tissue structure and function [1]. In contrast to actin filaments and microtubules, keratins are encoded by a large family of genes clustered at two divergent chromosomal sites: 17q21.2 (type I keratins, except K18) and 12q13.13 (type II keratins, including K18). These are also expressed in tissue and differentiation state-specific manner and play an important role in protecting epithelial cells from mechanical and non-mechanical stress and injury [2], [3], [4], [5].

Epithelial tumors continue to express keratins that are characteristic of their site of origin and therefore keratins are extensively used as immunohistochemical markers in diagnostic tumor pathology [3], [4]. Accumulating evidence points to the importance of keratins as prognostic markers and, more interestingly, as active regulators of epithelial tumorigenesis and treatment responsiveness [3]. Previous studies have reported alterations in keratin expression during oral carcinogenesis [6], [7], [8], [9]. Further, many keratins are recognized as independent markers of prognosis in OSCC [10], [11].

Within the oral cavity there is a complex pattern of keratin expression, reflecting both the type of epithelium and stage of differentiation specific expression. The basal proliferative layer of all oral epithelia expresses K5/K14 and K19. The suprabasal, differentiating layers of keratinized (cornified) epithelia express K1 and K10, while the differentiating layers of non-keratinized epithelia such as buccal mucosa and esophagus synthesize predominantly K4 and K13. Suprabasal epithelial cells of the hard palate and gingiva express K6, K16, and K76 [5], [12], [13], [14], [15]. Previous studies have reported altered terminal differentiation and keratin expression patterns in oral tumors, such as downregulation of K4, K5, K13 and K19 [11], [16], [17], [18], [19], [20], [21], [22], [23], [24], [25], [26], [27], [28]. Conversely, increased expression of K8/K18, K17 and K14 is reported in oral tumor tissues compared to the normal counterparts [6], [7], [8], [9], [10], [17], [18], [23], [29]. Various studies using *in-vitro* system have elucidated mechanistic role of keratins (K8/18, K19) in tumor invasion and metastasis [30], [31], [32]. However, *in-vitro* data may not fully reflect the *in-vivo* condition [33]. Interestingly, alterations of keratin expression pattern marks the common signature in human oral cancers and experimental oral tumors developed in animal models [34], [35]. Hence, we selected *in-vivo* model systems: the hamster model to demonstrate K76 downregulation during sequential progression of oral cancer, and the KO mice model to evaluate the effect of *KRT76* loss.

Gene expression analysis from our laboratory has revealed downregulation of *KRT76* in tumors of the oral cavity [26]. *KRT76*, a type II epithelial keratin (previously designated as K2p), is specifically expressed in the suprabasal cell layers of oral masticatory epithelium (the slightly orthokeratinized stratified squamous epithelium lining the gingiva and the hard palate) [13]. We now present data indicating that *KRT76* is downregulated prior to tumor development and its potential association with hyperproliferation in the formation of preneoplastic lesions.

## Materials and Methods

### Human Tissue Specimen Collection

The Institutional Review Board and the Local Ethics Committee of Tata Memorial Hospital (TMH) and Nair Hospital Dental College, approved the study. Written informed consents were obtained from all the study participants. Treatment naive neoprimary frozen tissues (n = 57) and paraffin embedded tissue blocks (n = 102) of different cohort of patients with gingivobuccal cancer (GBC) were obtained from the ICMR National Tumor Tissue Repository and Department of Pathology TMH, Mumbai respectively. Precancerous lesions (incident leukoplakia cases which are histopathologically hyperplastic lesions with focal mild to moderate dysplasia n = 61), independent normal tissues (n = 35), and inflamed tissues not associated with oral malignancy or pre-malignant conditions (n = 7) were collected from the Department of Oral Pathology, Nair Hospital Dental College, Mumbai; all these tissues were from gingivobuccal region. Tumor tissues with more than 70% tumor content were subjected to RNA extraction.

### Animal Models

The study on hamsters was conducted after approval from the Institutional Animal Ethics Committee (IAEC) of ACTREC, endorsed by the Committee for the Purpose of Control and Supervision of Experiments on Animals (CPCSEA), Government of India guidelines. Inbred male Syrian hamsters (6–8 weeks old; Animal house, ACTREC, India) were randomized (10 animals per group) and maintained under standard conditions: 22±2°C, 45% ±10% relative humidity, and 12-h light/dark cycle (7∶00 to 19∶00 light; 19∶00 to 7∶00 dark). The animals received an autoclaved standard pellet diet and plain drinking water *ad libitum.* Hamsters (3–5) were housed in the polypropylene cages provided with autoclaved rice husk bedding material available locally. The hamsters were topically treated with 7,12-dimethylbenz[α]anthracene (DMBA) (0.5%) in corn oil using a Gilson pipette (80 µl ≈ 0.4 mg) on their right buccal pouch, thrice a week for 16 weeks. The ‘corn oil’ was used for the treatment in vehicle control group. Animals in all groups were observed for apparent signs of toxicity such as weight loss or mortality during the entire study period. Following 1, 2, 4, 6, 8, 10, 12 and 16 weeks of DMBA applications, hamsters were euthanized (by CO_2_ chamber) 24 h after the last DMBA dose. Their buccal pouches were excised and fixed in 10% buffered formalin [36], [37].

The animal research ethical review committees of the Cancer Research UK Cambridge Research Institute and Cambridge University approved all the studies involving mice. *KRT76*-KO mice were obtained from the Wellcome Trust Sanger Institute (http://www.sanger.ac.uk/mouseportal/search?query=KRT76), and were maintained under the terms of a UK Government Home Office license 80/2378 (license holder Fiona M. Watt).

### RNA Isolation from Tissues

RNA was isolated from human tumor and normal tissues using the RNeasy mini kit (Qiagen, Germany) according to the manufacturer’s protocol. Briefly, 15–20 mg tissue was pulverized by grinding with liquid nitrogen, followed by addition of RLT buffer with β-mercaptoethanol (Sigma-Aldrich, USA). The homogenate was processed for column purification and isolation of RNA. DNA contamination was avoided by treating the column with RNase free DNase I (Ambion, USA). The quantity and quality of RNA was determined using Nanodrop ND-1000 (NanoDrop Technologies, Wilmington, DE, USA) and RNA 6000 Nano LabChip Kit on an Agilent 2100 Bioanalyzer (Agilent Technologies, CA) respectively.

### Quantitative Reverse Transcriptase-Polymerase Chain Reaction (qRT-PCR)

For complementary DNA (cDNA) synthesis, 1.5 µg of total RNA was reverse-transcribed with the High-Capacity cDNA Reverse Transcription Kit (Applied Biosystems, USA) following the manufacturer’s protocol. Twenty ng of cDNA were used for TaqMan qRT-PCR analysis and experiments were performed in duplicate (*KRT76* Assay Id: Hs00210581_m1, 18S RNA Assay Id: Hs99999901). Results were analyzed using SDS 2.3 and RQ manager software (Applied Biosystems). The relative expression of *KRT76* messenger RNA (mRNA) was determined using 18S ribosomal RNA as an endogenous control. These were compared between GBC cancers and unrelated normal tissues from the same site. The expression of *KRT76* in each sample was analyzed using the comparative CT method (also known as the 2^−ΔΔCT^ method) where ΔΔCT = [CT gene of interest − CT internal control (18S)] of test sample – [CT gene of interest − CT internal control (18S)] of reference sample. Fold change values for qRT-PCR data were calculated as 2^−ΔΔCT^ [38].

### Immunostaining of K76 in Human Oral Tissues

Formalin-fixed, paraffin-embedded GBC tissues (n = 102), OPLs (n = 61) and normal oral tissues (n = 21) were used for immunohistochemical (IHC) analysis. Five micron tissue sections were deparaffinized with xylene, rehydrated with sequential ethanol washes (100%, 90% and 70%). To quench the endogenous peroxidase activity, sections were incubated with 3% hydrogen peroxide in methanol for 30 min in dark. After heat based antigen retrieval with sodium citrate buffer (pH = 5.8), sections were incubated with normal horse serum. The sections were incubated overnight with rabbit polyclonal anti-human K76 antibody (1∶225, HPA019696, Sigma-Aldrich) at 4°C. For negative or isotype control, the primary antibody was replaced with rabbit serum used at respective antibody concentration. Sections were then incubated with biotinylated universal secondary antibody solution for 30 min followed by incubation with VectastainVR elite ABC reagent for the same time. The immunoreaction in tissue sections was visualized using 3,3′–diaminobenzidine tetrahydrochloridehydrate (Sigma-Aldrich). The slides were finally counterstained with hematoxylin and examined under microscope.

For immunofluorescence, deparaffinization and antigen retrieval steps were similar to those for IHC. Tissues were fixed in cold methanol for 10 min followed by blocking with 5% normal goat serum, 0.3% (v/v) Triton X-100 in PBS for 1 hr at room temperature. Tissues were next incubated with K76 antibody at a dilution of 1∶250 overnight at 4°C, followed by incubation with an Alexa Fluor 488 anti-rabbit antibody (Life technologies, USA) at 1∶200 dilution, for 1 hr at room temperature. Cells were counterstained with DAPI and viewed under a fluorescence microscope (Ziess; LSM-510 Meta Germany).

### Immunostaining of K76 in Animal Models

Formalin fixed hamster buccal pouch tissues were used from the following experimental groups for IHC analysis: 1) Control group: 1^st^, 2^nd^, 4^th^, 6^th^, 8^th^, 10^th^, 12^th^ and 16^th^ week hamsters buccal pouch topically treated with vehicle (no DMBA); 2) DMBA treated group: 1^st^, 2^nd^, 4^th^, 6^th^, 8^th^, 10^th^, 12^th^ and 16^th^ week hamsters buccal pouch topically treated with DMBA. Formalin fixed tissues from *KRT76*-Wild type (WT) and *KRT76*-KO mice were used for immumostaining and histopathological analysis. For experimental models, the IHC staining procedure was similar to that described earlier with minor changes in blocking, which was performed with 3% BSA and 2% goat serum; while secondary antibody was biotin conjugated anti- rabbit secondary raised in goat (Santa Cruz Biotechnology, USA).

### Immunohistochemical Assessment and Scoring

For assessment of K76 protein expression, the cytoplasmic staining intensity was categorized as 0 (absence of staining in any cell), +1 (weak staining in less than 10% of cells), +2 (moderate staining and/or 10 to 50% of positive cells), or +3 (strong staining in more than 50% cells) by pathologist (AP) (Figure S1). For further statistical analysis, the stained tissues were categorized in two groups: 0 and +1 as mild to no expression, while +2 and +3 as moderate to strong expression.

### Statistical Analysis

All statistical analyses were performed using IBM SPSS version 21. The Mann Whitney test was performed to analyze the difference between ΔCT values of tumor and normal samples obtained by qRT-PCR. The Chi-square test was used to determine the correlation between expression levels of K76 protein and tissue type, as well as clinicopathological characteristics. Polytomous logistic regression was used to evaluate the relationship of protein expression scores to the risk of OPL and OSCC development, with normal tissue as a reference; odds ratio (OR) were computed by adjusting for age and gender [22], [39]. Disease-specific survival (DSS) was calculated as the time from surgical diagnosis to the date of death due to cancer or to the last clinical follow-up prior to death. DSS was examined visually with Kaplan-Meier curves and analyzed by log rank tests. All p-values <0.05 were considered statistically significant.

## Results

### Patient Characteristics

The clinicopathological and demographic characteristics of all OPLs and tumor samples are summarized in Table 1. The patients in this study cohort were predominantly male tobacco habitués and tobacco chewing was the most prevalent habit. Most of the tumor samples were of moderate or poor grade, and mainly of pTNM stages III or IV. Approximately 50% of the cases showed lymph node invasion. Majority of OPLs had mild to severe hyperplasia and few showed presence of focal mild to moderate dysplasia.

**Table 1:** Demographic and clinicopathological characteristic of the study group.

Characteristics	qRT-PCROSCC(n = 57)†	IHC(n = 163)†	
		OSCC(n = 102)	OPL(n = 61)
Gender			
Males	40 (70%)	**80 (78.4%)**	55 (90.2%)
Females	17 (30%)	**22 (21.6%)**	6 (9.8%)
Age			
Median (IQR)#	52 (43.5–57.5)	**52 (41.7–64)**	45(34.5–56.6)
Habit profile			
Exclusive Chewers	46 (80.7%)	**34 (59.7%)**	19 (32.7%)
Exclusive Smokers	3 (5.3%)	**4 (7%)**	12 (20.7%)
Chewing andSmoking	8(14%)	**19 (33.3%)**	27 (46.6%)
Grade			
Well	2 (3.5%)	**12 (11.7%)**	–
Moderate	39 (68.4%)	**65 (63.7%)**	–
Poor	16(28.1%)	**25 (24.6%)**	–
Nodal involvement			
Negative (N0)	29 (50.9%)	**49 (48.0%)**	–
Positive (N+)	28 (49.1%)	**53 (52.0%)**	–
Stage (pTNM)			
I & II	3 (5.3%)	**13 (12.74%)**	–
III & IV	54 (94.7%)	**89 (87.26%)**	–

† Shown is the number of cases, except for Age,

# IQR: Interquartile range.

### Validation of Microarray Results by qRT-PCR

**Figure 1:**
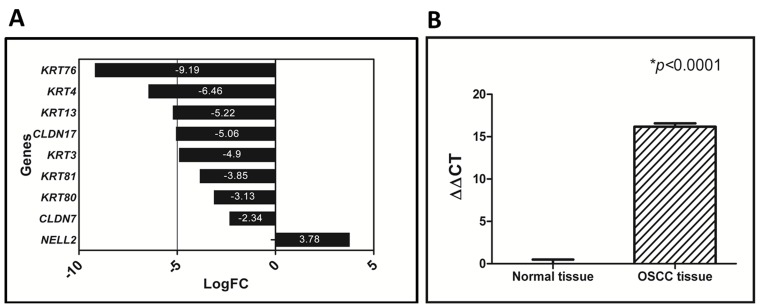
Downregulation of *KRT76* in GBCs. **A:** Data analyzed using GEO accession: GSE23558 demonstrate genes associated with structural molecular activity in GBCs. **B:** qRT-PCR analysis showed more than 15 fold downregulation of *KRT76* expression in tumors compared to normal oral tissue.

Microarray analysis of 27 GBC cases showed a significant downregulation of *KRT76*, as reported previously [26]. We observed downregulation of many genes associated with structural molecule activity Gene Ontology: 0005198 of which *KRT76* showed the highest fold change (Figure 1A). The Oncomine data source illustrated two more studies reporting consistent downregulation of *KRT76* in OSCC (Figure S2) [40], [41], [42]. To confirm the findings of the microarray analysis, we performed qRT-PCR using primers specific for *KRT76* in 57 OSCC and 14 normal tissues. qRT-PCR analysis revealed significant downregulation of *KRT76* RNA in tumor samples compared to normal samples (Figure 1B).

### Sequential Downregulation of K76 in Oral Carcinogenesis

**Figure 2:**
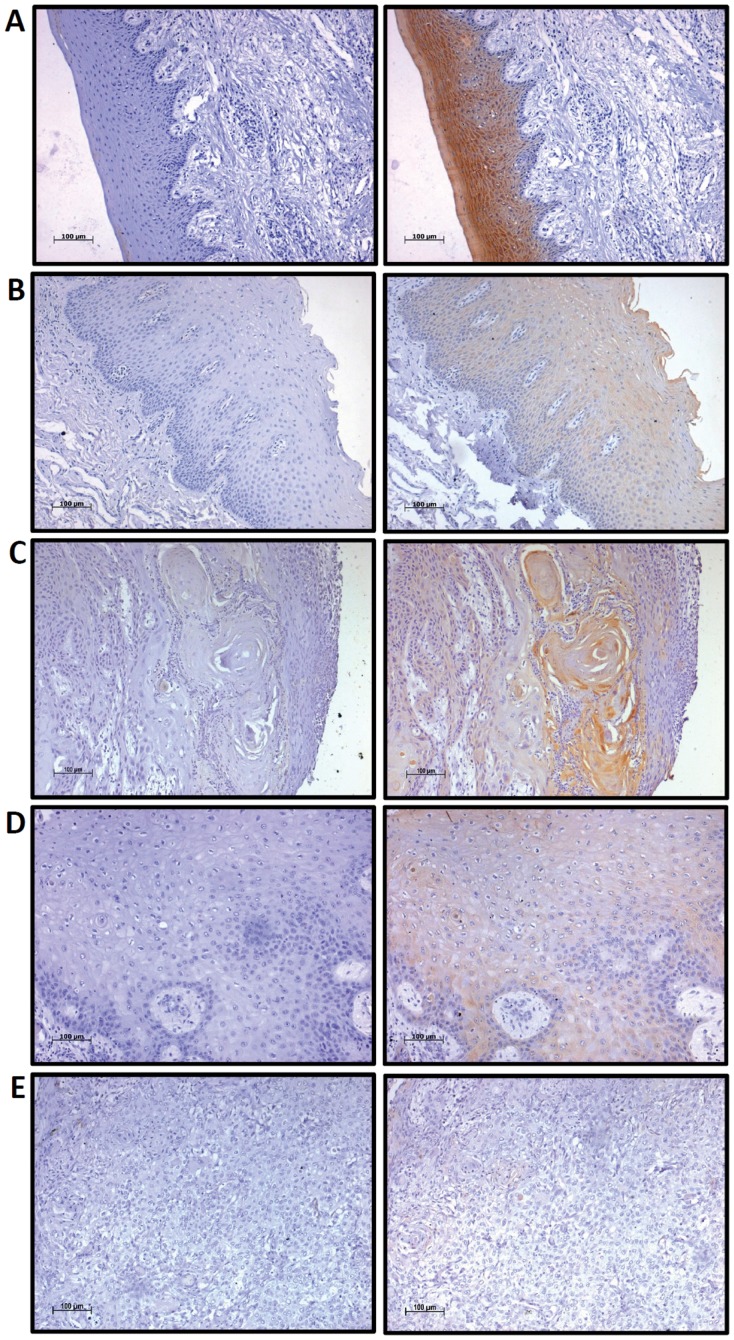
Immunohistochemical analysis of K76 expression in normal buccal mucosa, oral premalignant lesions and oral cancers. Representative IHC staining on **A:** Normal buccal mucosa, **B:** Oral Premalignant Lesions and OSCC (**C:** well differentiated, **D:** Moderately differentiated, **E:** poorly differentiated), with respective isotype control. Magnification 100X (Scale: 100 µm).

**Figure 3:**
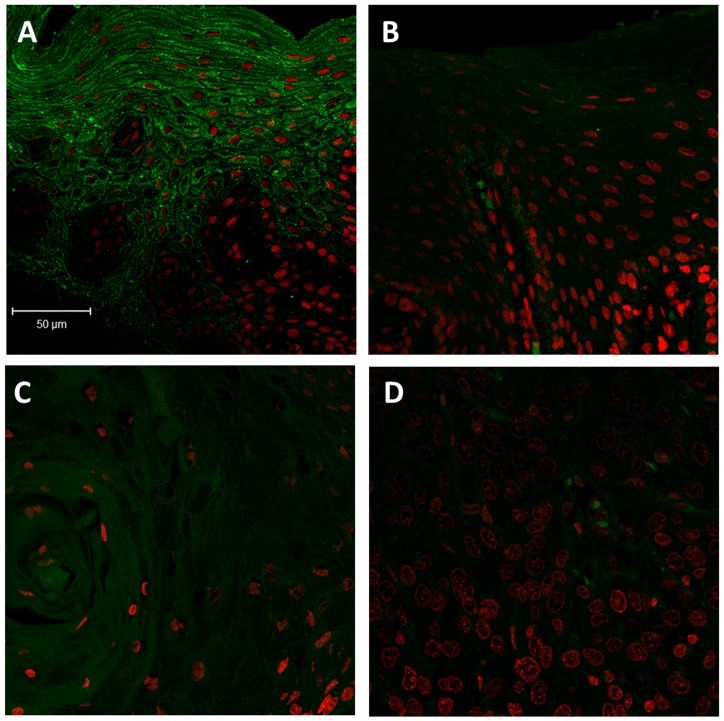
Immunofluorescence staining of K76 on human oral tissues. Representative Immunofluorescent staining of **A:** Normal oral tissue, **B:** OPL, **C:** Well differentiated tumor, **D:** Poorly differentiated tumor. K76 (Stained green, Alexa fluor 488), Nuclei stained with DAPI (pseudo red). Magnification 200X (Scale: 50 µm).

**Figure 4:**
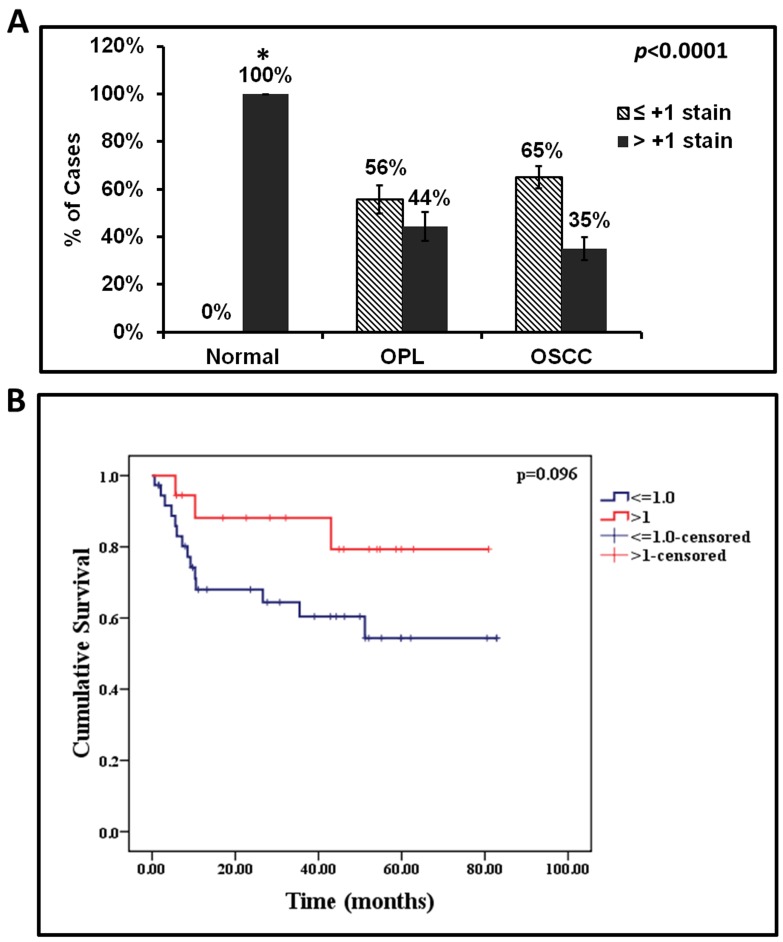
Correlation between loss of K76 expression with oral cancer development and patient survival. **A:** Significant downregulation of K76 was observed in OSCC and OPL compared to normal. **B:** Kaplan–Meier plot for DSS of gingivobuccal cancer patients with respect to K76 IHC staining intensity. Footnote: *All normals showed more than +2 grade stain.

K76 expression was analyzed in 184 oral tissues by immunohistochemistry (Figure 2). Normal gingivobuccal tissues expressed higher levels of K76 protein compared to OPL and invasive OSCC. Distribution of K76 expression was confirmed by immunofluorescence as illustrated in Figure 3. Normal oral epithelium showed K76 expression confined to the suprabasal, differentiating cell layers while, there was a gradual overall loss of K76 expression in OPLs and tumors. The frequency of K76 positive staining significantly decreased across the transition from normal tissue (100% positive) to OPL (44%) to oral tumor (35%) (Figure 4A).

To examine whether *KRT76* downregulation was associated with benign epithelial hyperproliferation (injured normal tissue without any association with oral preinvasive and invasive lesions), we performed IHC on inflamed buccal mucosa (n = 7). Even though these epithelia histologically appeared hyperproliferative, K76 staining was consistent with that seen in normal buccal epithelium (Figure S3). These results indicates that downregulation of *KRT76* expression is not associated with injury related proliferation and acute inflammation.

### Correlation of K76 Expression with Clinicopathological Parameters

Statistical analysis to determine the association of K76 expression and different clinical parameters, such as node, stage, grade, habit profile and outcome (recurrence and survival) was performed. Reduced expression of K76 showed a very weak association with survival (p = 0.096) (Figure 4B), whereas other parameters analyzed did not show any association. Polytomous Logistic regression with normal as the reference group showed a significant correlation of K76 downregulation with risk of developing OPL (p = 0.002) and OSCC (p≤0.0001) (Table 2).

**Table 2:** The effect of K76 expression loss with development of oral lesions.

K76 staining	Normal (n = 21)	OPL (n = 61)	OR	95% CI	*p value*	OSCC (n = 102)	OR	95% CI	*p value*
High(>1)	21	27	1	3.4–216.7	0.002	36	1	5.1–307	<0.0001
low(≤1)	0	34	27			66	40		

Polytomous logistic regression performed using normal as reference group indicated significant increase in risk of developing OPL and OSCC with decrease in staining.

### Loss of K76 Expression in an Experimental Model of Oral Carcinogenesis

**Figure 5:**
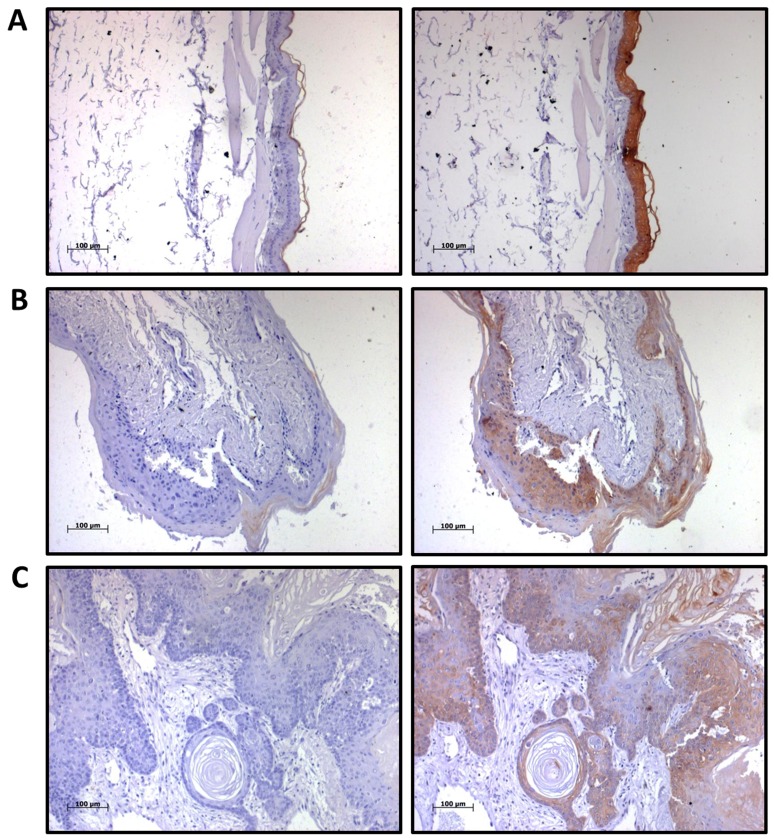
Expression of K76 in hamster model of oral carcinogenesis. IHC staining for K76 expression in hamster oral epithelium of **A:** Control group **B:** Hyperplastic lesion, **C:** Tumor with respective isotype controls. Magnification 100X (Scale: 100 µm).

K76 expression was analyzed by IHC in the buccal epithelium of DMBA treated hamsters (group details described in methods). Interestingly gradual decrease in staining intensity was observed with disease progression in hamster buccal epithelium (Figure S4). Irrespective of duration of treatment, control group showed higher levels of K76, while reduced expression was observed in premalignant lesions and oral tumors, which was similar to that seen in human hyperplastic lesions and OSCC (Figure 5).

### Mice Lacking *KRT76* Develop Hyperplastic Oral Lesions

**Figure 6:**
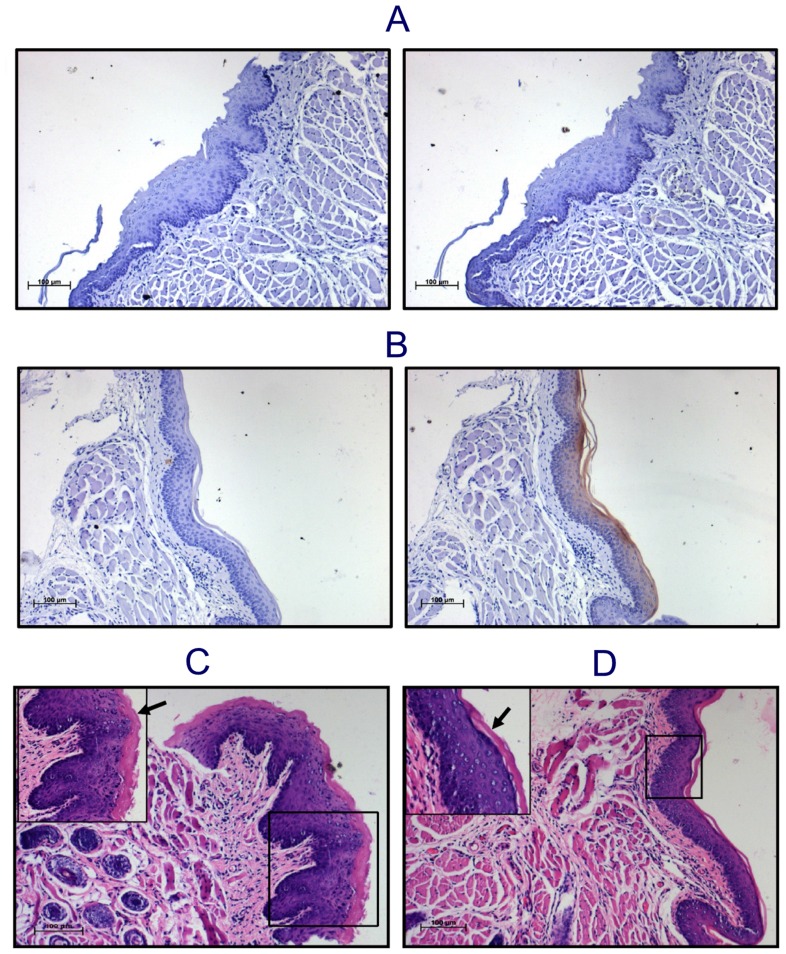
*KRT76*-KO mice show hyperplastic lesions in oral epithelium. K76 antibody specificity was determined by IHC on Oral epithelium of *KRT76*-KO mice which did not show any staining (**A**), whereas wild type mice of same strain showed moderate staining (**B**), with respective isotype control (left panel A & B). Histological observation of H & E stained buccal epithelium demonstrated hyperplastic changes and increased keratinization in KO (**C**)**,** compared to WT (**D**). Magnification 100X (Scale: 100 µm); selected area under 200×magnification.

To determine whether loss of *KRT76* is sufficient to induce premalignant lesions in the oral cavity, we examined the oral epithelia of *KRT76*-KO and *KRT76*-WT mice. Immunohistochemical analysis showed specific K76 staining in buccal epithelium of WT mice, whereas no staining was observed in *KRT76*-KO buccal epithelium, confirming specificity of K76 antibody (Figure 6 A, B). Histological examination of the buccal mucosa of *KRT76*-KO mice showed development of hyperplastic lesions along with increased keratinization across the epithelium, which was not observed in *KRT76*-WT mice (Figure 6 C, D). In contrast, the epithelium of the dorsal tongue, which is normally *KRT76*-negative, exhibited normal homeostasis in *KRT76-*KO mice indicating that *KRT76* loss associated abnormalities are highly sub-site specific in oral cavity (Figure S5). However, none of the *KRT76*-KO mice in the entire life span developed spontaneous oral tumors.

## Discussion

Deregulated keratin expression is associated with impaired epithelial differentiation and organization during OSCC progression [4], [15], [21], [24], [41], [43], [44]. Our microarray based gene expression profile of 27 advanced stage gingivobuccal cancers previously revealed deregulation of several keratins, namely *KRT4, KRT13, KRT19, KRT76*, which are normally expressed in the oral cavity. *KRT76* was found to be the topmost downregulated gene amongst all differentially expressed genes [26]. Gene expression profiles of oral cancer obtained by other groups have also shown consistent downregulation of *KRT76* [21], [40], [41]. We now report, for the first time, differential expression of *KRT76* in human and hamster oral precancerous and cancerous lesions, and show that loss of *KRT76* is sufficient to cause hyperplasia in the oral cavity of the mice.

We validated our previous microarray findings in an independent patient cohort by qRT-PCR and IHC; both these techniques showed reduced expression of *KRT76*. While previous reports have demonstrated changes in keratin gene expression associated with severe dysplasia and poorly differentiated SCC, reflecting gross changes in epithelial differentiation and maturation [43], [44], our studies are the first to indicate that loss of a specific keratin is sufficient to initiate preneoplastic changes. We did not find association of K76 downregulation with clinicopatholgical parameters such as node, grade, clinical outcome; nor with benign inflammation-associated hyperproliferation. Although, the fact that K76 downregulation is observed in leukoplakia, a preinvasive oral lesion and is sustained during the development of frank malignancy, indicates its association with the early stages of oral carcinogenesis.

Interestingly, we observed gradual decrease in K76 expression during the sequential process of tumor development in DMBA treated buccal epithelium of hamster (Figure S4). The K76 downregulation was consistent with human OPL and OSCC. Although hamster cheek pouch model has several areas of uniqueness, it also lacks lymphatic drainage as observed in humans, mice, or rats, which makes it immunoprotected [33], [45], [46]. However none of the existing animal models in studies on oral cancer are fully satisfactory and simulate tobacco chewing [33], [47], [48]. Hamster is one of the extensively used models, as the oral epithelium has similar histological and genetic events involved in the development of premalignant lesions and tumors as in humans [34], [49], [50], [51].

In order to investigate the effect of *KRT76* loss, we used *KRT76*-KO mice. The transgenic and knockout mouse models provide unique advantage of genetic manipulation of specific target gene/s, it also has similar intracellular signaling pathways as of humans [52]. In-vivo systems over comes the weakness of in-vitro experiments which fails to replicate the complex cellular and tissue interaction in an organism; hence, better suited for observing the overall effects of a target gene in a living system. *KRT76*-KO mice displayed hyperplastic changes in buccal epithelium, however they do not spontaneously develop tumors similar to previous reports on other keratin knockout mice models [53], [54], [55]. Our current findings suggest that the loss of *KRT76* may not be a sole molecular event leading to oral cancer development. However, the hyperplastic changes observed in *KRT76*-KO mice points to an indirect role of *KRT76* in regulating proliferation of the basal layers of buccal mucosa similar to previous findings of *KRT10* loss [54]. Overall, our data implies the fact that carcinogenesis being multifactorial and multistep process, potential role of *KRT76* as one of the factor, which alone is not sufficient for cell transformation; however, its contribution in oral carcinogenesis cannot be ruled out.

We envision a number of possible ways in which *KRT76* loss contributes to cancer development. One is that it contributes to a barrier defect in the epithelium, which may render the tissue more susceptible to penetration by carcinogens [56]. Another is that *KRT76* loss may lead to a disturbed inflammatory infiltrate; which is observed in human and mouse epidermis on loss of structural proteins [57], [58]. We did not see loss of *KRT76* in benign hyperproliferative oral epithelium, with associated inflammation, nevertheless, altered immune infiltrates are a hallmark of OSCC [59], [60].

Future investigations are needed to assess the impact of *KRT76* loss in predicting high-risk precancerous lesions of oral cavity. We observed *KRT76* downregulation in patients with gingivobuccal cancers – a sub site of oral cancer, which is etiologically associated with peculiar tobacco and betel quid chewing habit common in India. These results have to be generalized with caution to other etiologies associated with development of oral tumors. Although, *KRT76* loss is characteristic of gingivobuccal tumors it is not associated with cell transformation, our results warrant future studies to understand other key players driving the process of oral carcinogenesis.

## Supporting Information

Figure S1 **Representative images of IHC grades.** Manual grading of IHC staining was done as 0, +1, +2, +3 depending on staining intensity.
(TIF)

Figure S2 **Oncomine data search for** ***KRT76*** **expression in Oral tissues.** Data search showed two studies reporting *KRT76* downregulation; A: Ginos et.al Cancer Res. 2004 Jan 1;64(1): 55–63; Observed fold change of about −24.55, and it ranked in top 10% of under expressed genes. B**:** Toruner GA et.al Cancer Genet Cytogenet. 2004 Oct 1;154(1): 27–35; Observed fold change of about −69.55, and it ranked in top 14% of under expressed genes.
(TIF)

Figure S3 **Expression of K76 in inflamed buccal mucosa.** IHC staining of inflamed buccal epithelium showed higher expression of K76 with respective isotype control.
(TIF)

Figure S4 **Sequential downregulation of K76 expression during tumor development in hamster buccal epithelium.** Gradual decrease in K76 IHC staining was observed in different weeks, [1^st^ week (**B**), 2^nd^ week (**C**), 4^th^ week (**D**), 6^th^ week (**E**), 8^th^ week (**F**), 10^th^ week (**G**), 12^th^ week (**H**), 16^th^ week (**I**)], of DMBA treated buccal epithelium; whereas controls of all weeks showed consistent staining,(**A**).
(TIF)

Figure S5 **Histology of KO (A) and WT (B) mice dorsal tongue, along with respective K76 IHC staining.**
(TIF)
