# Supplementary figures and images for: Downregulation of Keratin 76 Expression during Oral Carcinogenesis of Human, Hamster and Mouse
Source: PLoS One. 2013 Jul 30;8(7):e70688. doi: 10.1371/journal.pone.0070688 (PMC3728316; doi:10.1371/journal.pone.0070688)

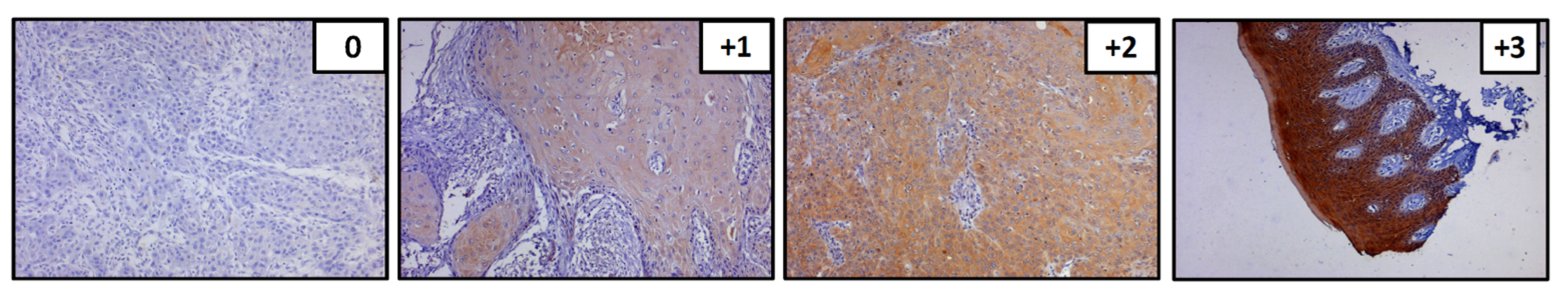

Supplement: Figure S1 — Representative images of IHC grades. Manual grading of IHC staining was done as 0, +1, +2, +3 depending on staining intensity. (TIF) [file pone.0070688.s001.tif]

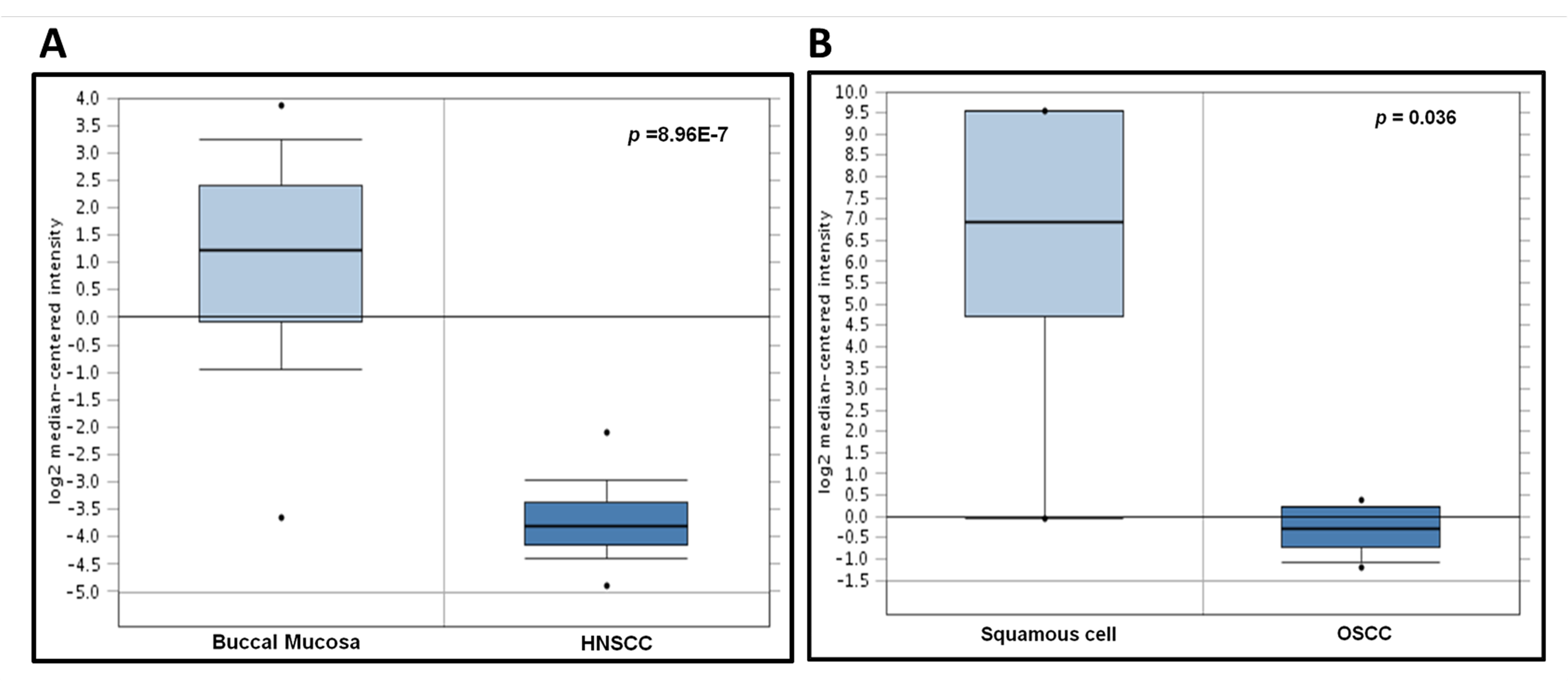

Supplement: Figure S2 — Oncomine data search for KRT76 expression in Oral tissues. Data search showed two studies reporting KRT76 downregulation; A: Ginos et.al Cancer Res. 2004 Jan 1;64(1): 55–63; Observed fold change of about −24.55, and it ranked in top 10% of under expressed genes. B: Toruner GA et.al Cancer Genet Cytogenet. 2004 Oct 1;154(1): 27–35; Observed fold change of about −69.55, and it ranked in top 14% of under expressed genes. (TIF) [file pone.0070688.s002.tif]

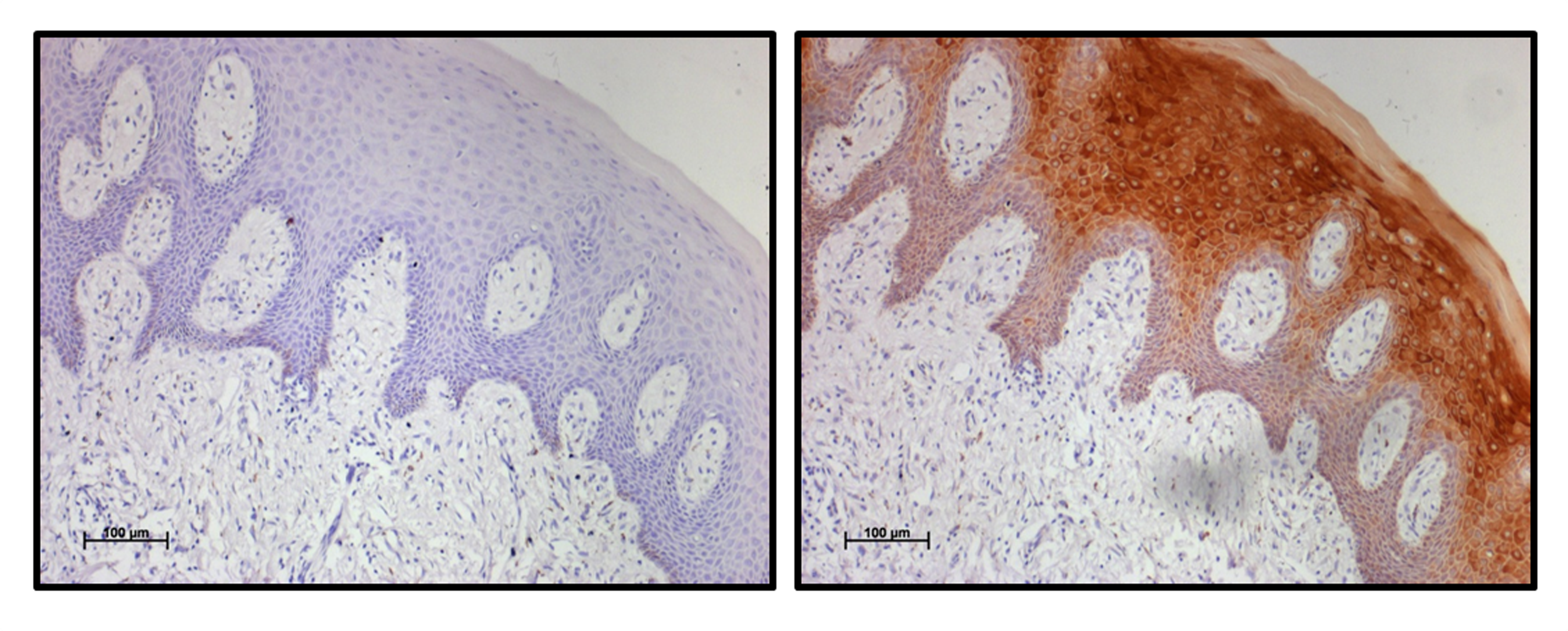

Supplement: Figure S3 — Expression of K76 in inflamed buccal mucosa. IHC staining of inflamed buccal epithelium showed higher expression of K76 with respective isotype control. (TIF) [file pone.0070688.s003.tif]

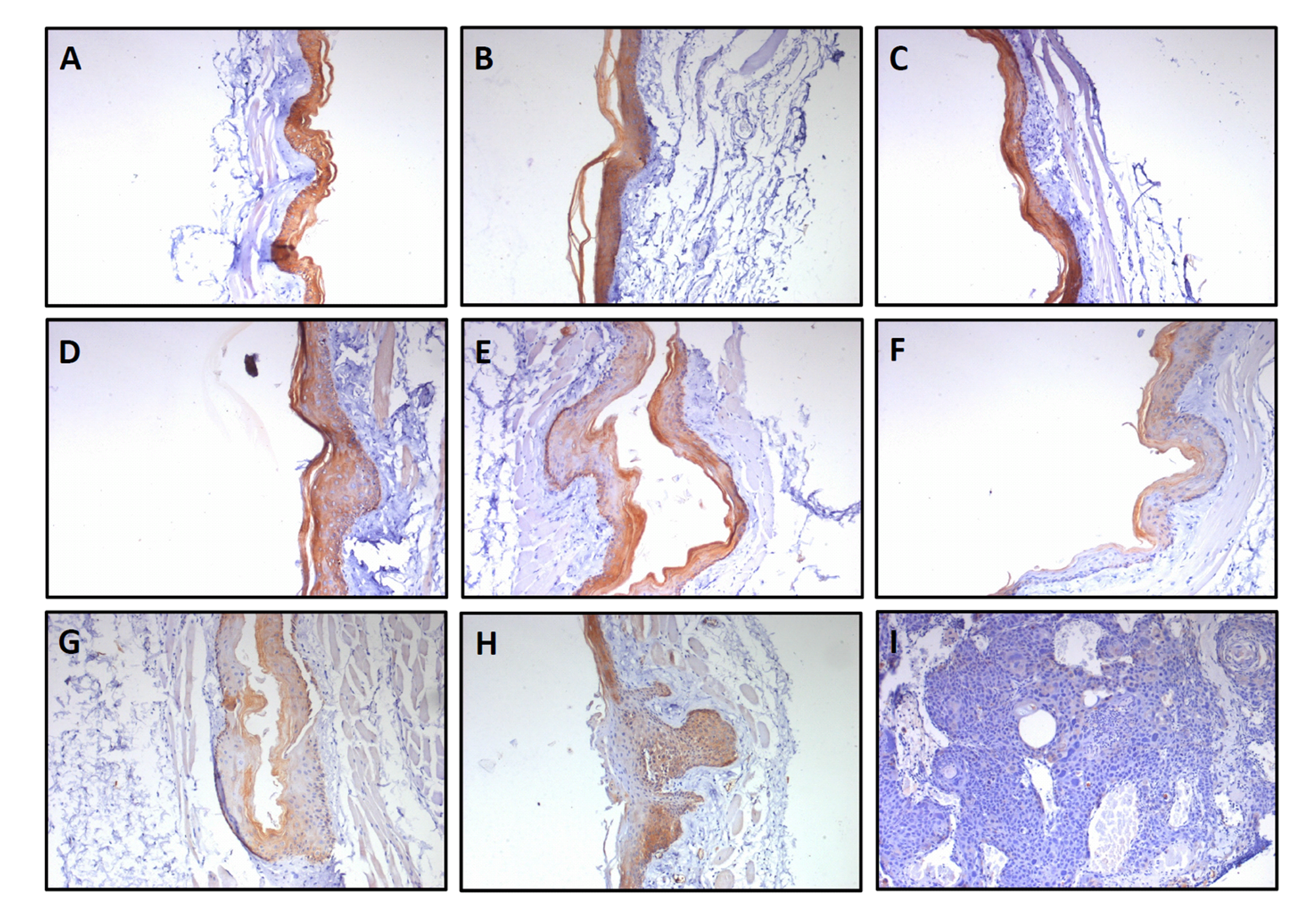

Supplement: Figure S4 — Sequential downregulation of K76 expression during tumor development in hamster buccal epithelium. Gradual decrease in K76 IHC staining was observed in different weeks, [1st week (B), 2nd week (C), 4th week (D), 6th week (E), 8th week (F), 10th week (G), 12th week (H), 16th week (I)], of DMBA treated buccal epithelium; whereas controls of all weeks showed consistent staining,(A). (TIF) [file pone.0070688.s004.tif]

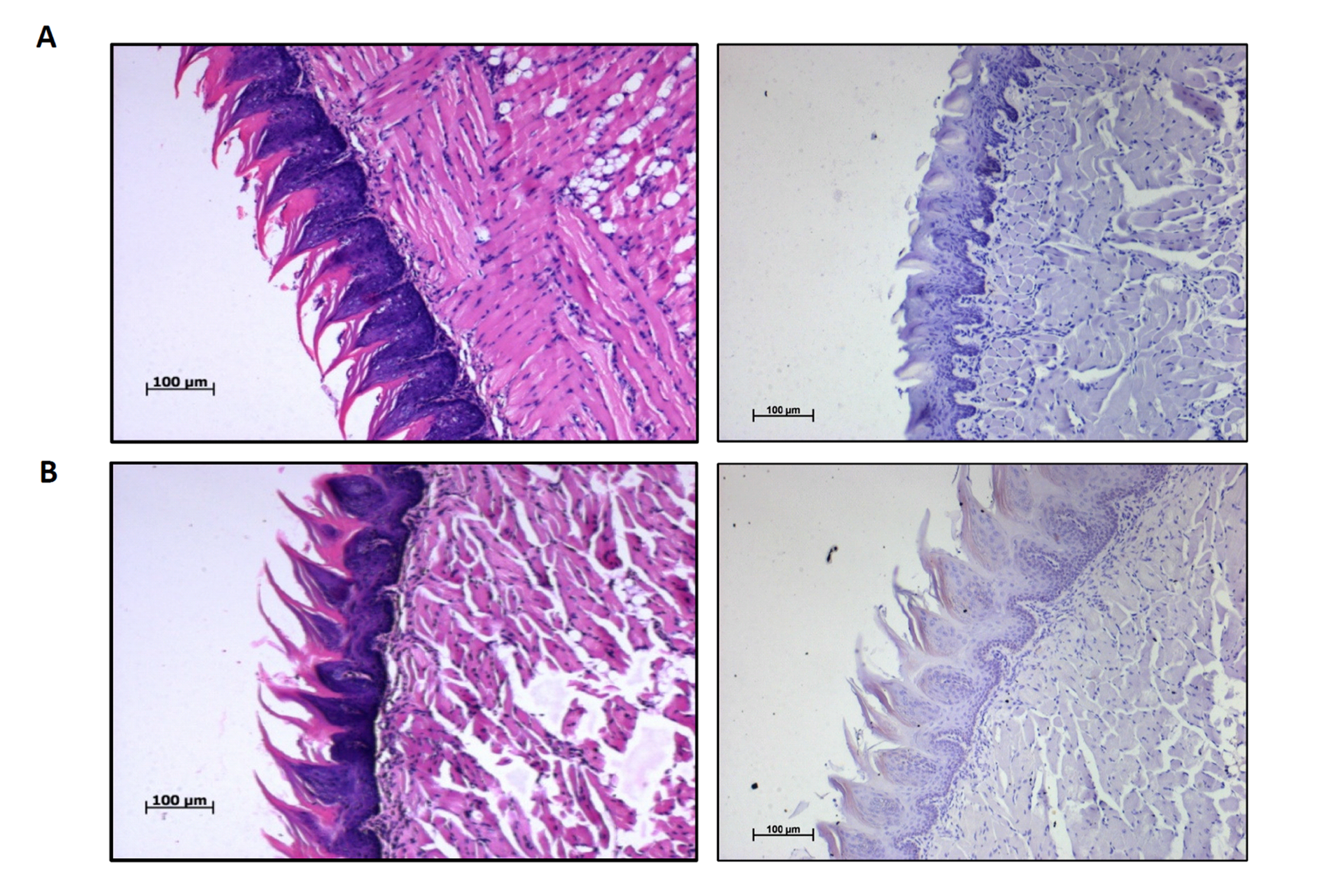

Supplement: Figure S5 — Histology of KO (A) and WT (B) mice dorsal tongue, along with respective K76 IHC staining. (TIF) [file pone.0070688.s005.tif]
